# Amyloid Beta in Alzheimer's Disease: Mechanisms, Biomarker Potential, and Therapeutic Targets

**DOI:** 10.1002/cns.70688

**Published:** 2025-12-09

**Authors:** Shamseddin Ahmadi, Shiler Khaledi, Kimia Ahmadi, Kambiz Hassanzadeh

**Affiliations:** ^1^ Department of Biological Science, Faculty of Science University of Kurdistan Sanandaj Iran; ^2^ Student Research Committee, School of Medicine Tabriz University of Medical Sciences Tabriz Iran; ^3^ Robert Wood Johnson Medical School, Institute for Neurological Therapeutics, and Department of Neurology Rutgers Biomedical and Health Sciences Piscataway New Jersey USA

**Keywords:** amyloid plaques, APOE, blood‐based biomarker, lecanemab, neurodegenerative disorders, neuroinflammation, post‐translational modifications

## Abstract

**Main Problems:**

The accumulation of amyloid beta (Aβ) plaques and neurofibrillary tangles (NFTs) composed of Tau protein is two characteristic brain pathologies in Alzheimer's disease (AD). However, the Aβ hypothesis has recently faced challenges due to the limited clinical efficacy of anti‐Aβ antibodies, such as aducanumab and lecanemab.

**Methods:**

This comprehensive review highlights recent advances and debates regarding the pathophysiology of Aβ peptides and plaques in AD, as well as their use as biomarkers and drug targets.

**Results:**

Aβ aggregation is primarily driven by an imbalance between its generation from amyloid precursor protein (APP) and its clearance from the brain, processes influenced by various risk factors. The toxicity of amyloid plaques is affected by the accumulation of different Aβ species with varying lengths and post‐translational modifications of Aβ. Additionally, pathways including neuroinflammation, blood–brain barrier deterioration, autophagy and mitochondrial dysfunction, lipid raft changes, and oxidative stress have pivotal roles in AD. Therefore, a clear map of Aβ's upstream regulators and downstream effectors is crucial for developing effective diagnostics and treatments for AD.

**Conclusions:**

Incorporating new research findings and ongoing debates surrounding the Aβ cascade hypothesis is crucial for improving early diagnosis and for guiding the development of effective treatments for AD.

## Graphical abstract

The limited clinical efficacy of anti‐Aβ antibodies has challenged the Aβ hypothesis, compelling the integration of key mechanisms—including neuroinflammation, mitochondrial dysfunction, oxidative stress, and post‐translational modifications of Aβ—into a revised model of Alzheimer's disease.

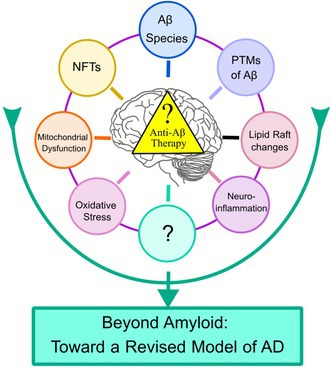

## 1 Introduction

Alzheimer's disease (AD) is a progressive neurodegenerative condition classified as dementia, characterized by the progressive deterioration of memory and cognitive function [1, 2]. Although AD rarely presents in mid‐life, it predominantly affects the elderly, making age a major risk factor for the disease. The exact onset of clinical AD is difficult to ascertain, but subtle and intermittent episodic memory problems are often the earliest symptoms. As the disease progresses, it shifts from declarative to non‐declarative memory impairment, affecting a broad range of cognitive and behavioral processes [2, 3]. AD significantly impacts patients' quality of life and imposes a substantial emotional and financial burden on patients, caregivers, and society as a whole [3, 4]. With increasing life expectancy and an aging population, its prevalence is projected to rise significantly by 2050 [3]. Although there has been notable progress in understanding the mechanisms underlying the disease, effective treatments to halt its progression remain unavailable. Given the significant clinical and financial burden of the disease, there is an urgent need for more effective treatments. Achieving this requires a deeper understanding of the molecular and cellular pathways involved, which could enable early detection and the development of better treatment options.

The early stage of the disease is believed to result from the age‐related production of a protein called amyloid beta (Aβ) from the amyloid precursor protein (APP) and impaired Aβ clearance in the brain. This Aβ pathology is thought to trigger a cascade of events that ultimately produces the AD phenotype [5, 6]. However, a growing body of research underscores the significant contribution of Aβ‐independent mechanisms to the onset and progression of AD [7]. Therefore, advancing beyond the traditional Aβ cascade hypothesis by integrating new insights into AD pathogenesis is crucial for a comprehensive understanding of the disease and the development of strategies to slow its progression [8].

This review explores the dual physiological and pathological roles of Aβ, focusing on its interactions with cellular and molecular alterations in AD. It begins by summarizing Aβ's significance in disease progression and its growing utility as a diagnostic biomarker, then provides a critical appraisal of current and emerging Aβ‐targeting therapies, weighing their respective promises and limitations. Finally, the review proposes a pathogenic framework that extends beyond Aβ and Tau pathology and outlines essential future directions for the field.

## 2 Pathological Features and Etiology of AD

AD is characterized by distinct pathological hallmarks at both macroscopic and microscopic levels. Macroscopically, the AD brain exhibits macroscopic neurodegenerative changes such as ventricular enlargement, reduced brain volume due to gray matter atrophy, and abnormalities in white matter [9, 10, 11]. Pathologically, AD is defined by Aβ plaques, composed of fibrillary aggregates of Aβ peptides, and neurofibrillary tangles (NFTs) formed by hyperphosphorylated Tau protein. These pathological features ultimately contribute to neuronal degeneration and cognitive impairment in AD [2]. AD is additionally characterized by astrogliosis and microgliosis [12, 13], as well as changes in signaling pathways associated with synaptic plasticity and glutamate receptors [14]. Other neuropathological features of AD include dystrophic neurites, dendritic spine loss, accumulation of abnormal endosomes, lysosomes, and mitochondria, as well as glia‐mediated inflammation [15]. Furthermore, cerebral amyloid angiopathy (CAA), characterized by Aβ fibrils deposition in the brain vessels, is commonly observed in AD [4, 16, 17]. Another feature seen in AD is granulovacuolar degeneration (GVD), first described by Teofil Simchowicz in 1911 [18], which involves granule‐containing vacuoles accumulating in hippocampal neurons, particularly in CA1, pre‐subiculum, CA2, CA3, and CA4 areas, eventually spreading to other brain regions [19, 20]. The measurement of these microscopic pathological alterations offers insights into the progression of the disease and symptoms [16].

AD is classified into two main types, familial AD and sporadic AD. Familial AD, which accounts for 5 to 10% of AD patients, is caused by genetic mutations and is often referred to as early‐onset AD (EOAD) [21]. It is mainly associated with mutations in the presenilin 1 and 2 or APP genes, which lead to abnormal processing of APP and an increased production of toxic Aβ species [21, 22]. In contrast, sporadic AD, also known as late‐onset AD (LOAD), constitutes the majority of cases (90%–95%) [23, 24]. It is largely attributed to aging‐related processes and impaired Aβ clearance mechanisms including proteolytic enzymes, chaperone molecules like apolipoprotein E (APOE) and clusterin, and degradation pathways such as autophagy and the proteasome system [22, 23]. While sporadic AD is less frequently linked to hereditary factors, it is nevertheless linked to genetic alterations. The presence of the ε4 variant of the APOE (APOE ε4) in 10%–20% of populations is a notable risk factor for sporadic AD. Apart from genetic influences, several environmental and lifestyle factors have been implicated in the development of sporadic AD, including vascular disease, traumatic brain injury, risk factors associated with diet, metal exposure, infection and hyperactivation of the immune system, and mitochondrial dysfunctions [25]. Interestingly, while Aβ accumulation is associated with cognitive decline, approximately 25%–30% of amyloid‐positive older adults maintain normal cognitive performance, a phenomenon attributed to cognitive reserve, which is often linked to higher levels of education and occupational engagement [26]. Considering the aforementioned data along with new findings from AD animal models and clinical data [27, 28], Aβ may not be the only underlying cause of AD. Therefore, Aβ‐independent molecular pathways in AD pathogenesis should be considered. It seems that integrating these diverse pathological and etiological insights is fundamental to unraveling the complexity of AD and translating this knowledge into successful therapeutic interventions.

## 3 Amyloid Precursor Protein (APP)

### 3.1 APP Structure and Processing Pathways

The *APP* gene is located on chromosome 21 and encodes the amyloid precursor protein (APP), a member of the amyloid precursor‐like proteins (APLP) family. APP is classified as a single‐pass transmembrane protein and is characterized by its substantial extracellular N‐terminal domain, which exhibits biological activity. The Aβ sequence is primarily situated within the extracellular N‐terminal region. In contrast, the shorter intracellular C‐terminal region contains a conserved amino acid sequence known as YENPTY (Tyr‐Glu‐Asn‐Pro‐Thr‐Tyr) [29, 30, 31]. The APP mRNA undergoes alternative splicing, generating eight isoforms, with three being the most prevalent. The 751 and 770 amino acid isoforms of APP are predominantly expressed in various peripheral tissues and fibroblasts, whereas the 695 amino acid isoform is mostly expressed in the central nervous system (CNS), with particularly high expression in the cortex and hippocampus [32, 33]. While all three major APP isoforms are expressed in cultured astrocytes and microglia [34], their expression in glial cells in vivo has not been reported [35].

**FIGURE 1:**
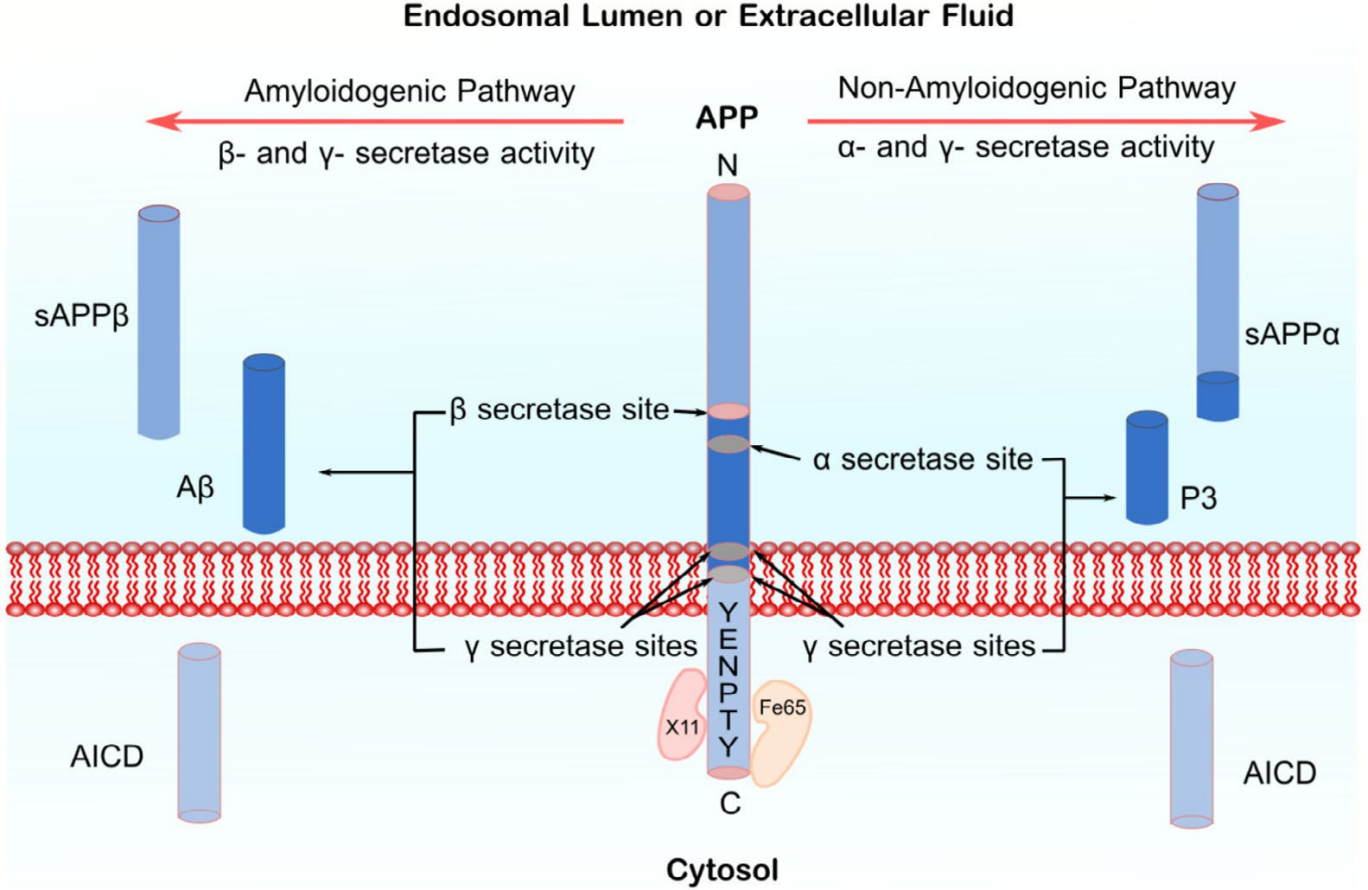
Amyloidogenic and non‐amyloidogenic pathways. APP is a single‐passed membrane protein consisting of an extracellular N‐terminal domain, a membrane‐spanning domain, a YENPTY sequence, and an intracellular C‐terminal domain. In the amyloidogenic pathway, the APP molecule is cleaved by β‐secretase (BACE1) and γ‐secretase, leading to the production of soluble APP β (sAPPβ), Aβ, and an intracellular C‐terminal fragment (AICD) fragment. In the non‐amyloidogenic pathway, APP is cleaved first by α‐secretases and subsequently by γ‐secretases producing soluble APPα (sAPPα), the P3 fragment, and an identical AICD.

APP undergoes cleavage through two distinct processing mechanisms: the amyloidogenic pathway and the non‐amyloidogenic pathway, generating molecular fragments that are either released into the extracellular space, retained within the cell or linked to the cell membrane [30, 36]. In the non‐amyloidogenic processing, APP is initially cleaved by members of the α‐secretase family, such as A Disintegrin And Metalloproteinase domain‐containing protein 10 (ADAM10) and ADAM17, producing a soluble ectodomain fragment called sAPPα, and a membrane‐bound 83 amino acid fragment known as C83. Subsequent cleavage of the C83 segment by γ‐secretase generates a P3 fragment and an APP intracellular C‐terminal domain (AICD) that is released into the cytoplasm. In contrast, the amyloidogenic pathway is initiated by enzymes called β site APP cleaving enzyme 1 and 2 (BACE1 and BACE2), resulting in the release of the soluble extracellular portion of APPβ (sAPPβ) and a membrane‐bound 99 amino acid fragment (C99). The cleavage of C99 by γ‐secretase produces the amyloidogenic Aβ peptide and an AICD fragment identical to that formed in the non‐amyloidogenic pathway [4, 30, 32, 33] (Figure 1). Together, sAPPα and sAPPβ account for at least 50% of total APP in the brain. Despite their structural similarity—with sAPPα being 16 amino acids longer than sAPPβ—they exhibit distinct physiological functions [32]. These differences raise critical questions regarding the fundamental physiological and pathophysiological roles of APP.

In addition to the canonical processing pathways, there are non‐canonical APP‐processing pathways, including the delta (δ)‐pathway, eta (η)‐pathway, meprin‐pathway, and APP‐cleavage pathway by caspase. In the caspase pathway, a C31 fragment is produced, which may contribute to neuronal apoptosis. Moreover, caspase activity in combination with γ‐secretase generates a small peptide called Jcasp, thought to inhibit presynaptic transmitter release [32]. A detailed discussion of non‐canonical APP‐processing pathways and their products falls beyond the scope of this review. However, in light of the heterogeneity of AD phenotypes and ongoing debates concerning the roles of Aβ oligomers and plaques, we propose that further investigation into the products of non‐canonical APP processing and their potential interactions with canonical Aβ peptides is warranted in AD pathogenesis.

### 3.2 APP Biosynthesis and Aβ Production in Neurons

The secretory pathway is responsible for the synthesis and trafficking of the APP molecule, which translocates from the endoplasmic reticulum to the cellular membrane. While APP is predominantly localized within the Golgi complex, a minor fraction of APP is found on the cell membrane. The majority of APP on the cell surface is mostly internalized by the cell through early endosomes, where a portion is recycled back to the plasma membrane, while the rest is directed toward lysosomes for degradation [37, 38]. Both α‐secretases (ADAM 9, 10, 17, 19) and BACE1 are present on the cell surface and in the trans‐Golgi network (TGN), with ADAM10 being the predominant α‐secretase in neurons. α‐secretase is primarily found on the cell surface, whereas BACE1 is mainly found in the TGN and endosome. The endosome maintains an ideal acidic pH for BACE1 function, suggesting that amyloidogenic APP processing takes place in endosomes [39]. A critical distinction in APP processing is dictated by the predominant localization of γ‐secretase to intracellular compartments. This leads to the non‐amyloidogenic cleavage of APP at the cell surface, in contrast to the amyloidogenic processing of APP within endosomes and the TGN [38, 39]. The APP protein is synthesized and metabolized extensively in neurons. Multiple proteolysis mechanisms regulate APP processing, with certain pathways resulting in the formation of Aβ peptide [33].

**FIGURE 2:**
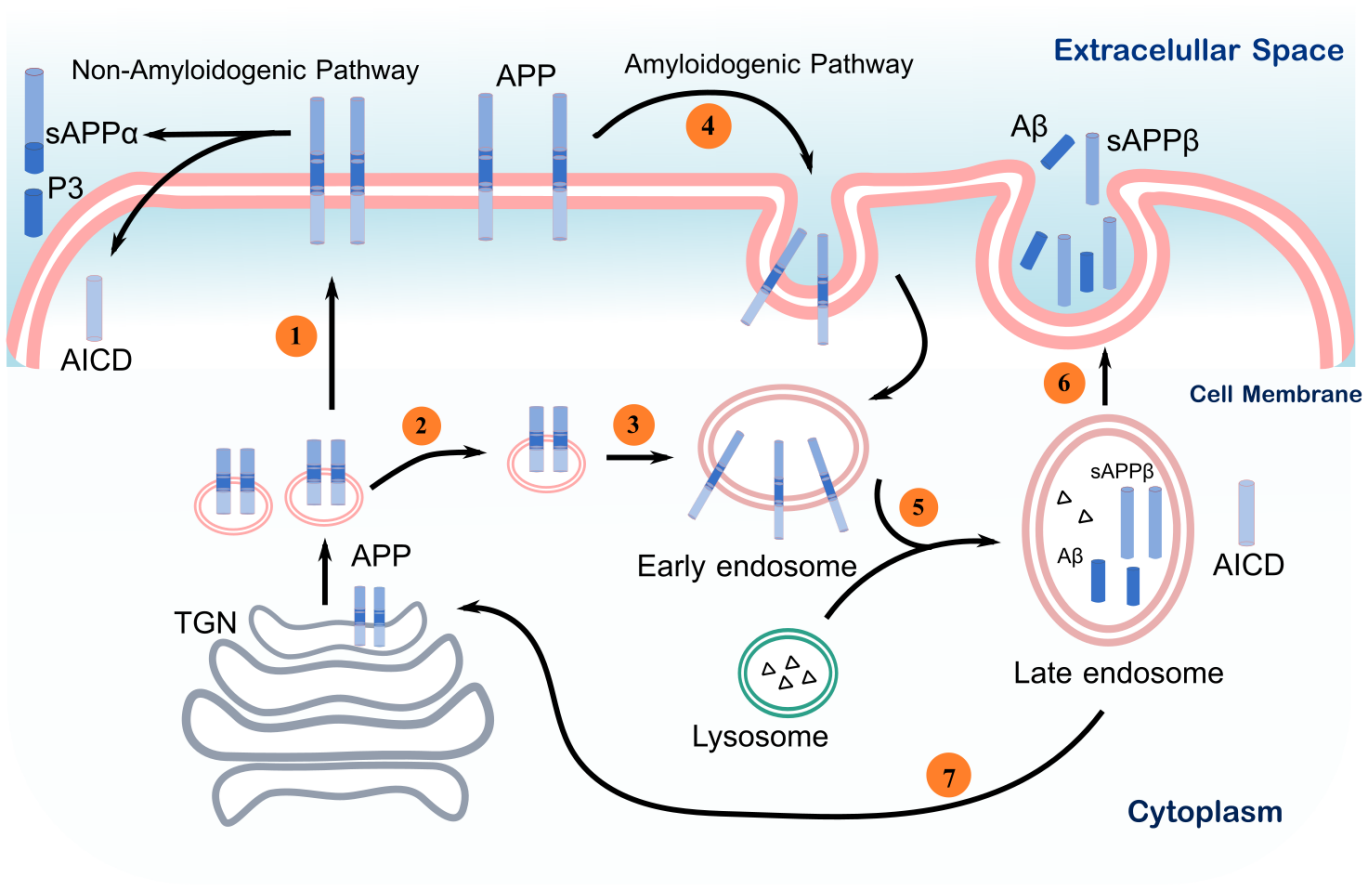
APP synthesis, trafficking, and processing in neurons. APP molecules are synthesized in the rough endoplasmic reticulum and are transported to the Golgi apparatus. From the trans‐Golgi network (TGN), APP can either be directed to the cell surface (1) or sent directly to the endosomal compartment (2). At the cell surface, APP is immediately processed by α‐secretase and subsequently by γ‐secretase in the non‐amyloidogenic pathway. The process continues with the internalization of additional APP molecules into clathrin‐coated pits (4), where they are processed by BACE1 and γ‐secretase leading to the production of Aβ, which is subsequently released into the extracellular area (5 and 6). Lysosomes are cellular organelles that break down and recycle vesicles. A portion of endosomal compartments is retrogradely transported to the TGN, facilitated by retromer molecules (7).

The APP protein is synthesized within the endoplasmic reticulum and Golgi complex, before being transported to neuronal axons. From there, it is further conveyed to synaptic terminals. The newly produced APP originating from the TGN can be transported either to the cell surface or directly into the endosomal compartment. Clathrin‐coated vesicles mediate both of these pathways. The surface pool of APP is determined by the combined effects of secretory trafficking, internalization, and the efficiency of secretase processing [40]. Hence, only a limited number of APP molecules are present on the cellular membrane at any given time. A portion of the substance undergoes direct processing by β‐secretase, followed by γ‐secretase, which does not result in the production of Aβ [22, 41]. The clathrin‐coated pit facilitated the internalization of additional APP molecules into distinct endosomal vesicles where they interact with BACE1 and γ‐secretase. As a result, Aβ is generated and subsequently released into the extracellular space. Lysosomes within the cell are responsible for the degradation and recycling of vesicles. The endosomal compartment and the TGN have a retrograde connection, which is facilitated by retromer molecules [33, 36, 41] (Figure 2).

Whereas the ADAM family with α‐secretase activity is widely expressed, the BACE1 enzyme is particularly concentrated in neurons. Research confirms that BACE1, but not BACE2, is a key regulator of Aβ synthesis, as evidenced by the decreased Aβ production following a reduction in BACE1 expression [42]. The γ‐secretase enzyme is a multiprotein complex composed of several subunits. It consists of two catalytically active proteins, presenilin1 and presenilin2, as well as a transmembrane glycoprotein type 1 known as nicastrin. Additionally, it contains two multipass transmembrane proteins, known as anterior pharynx defective (Aph‐1) and presenilin enhancer2 (Pen‐2). These proteins are responsible for the cleavage of integral membrane proteins, including Notch receptor and APP. The cell surface enzyme γ‐secretase completes α‐secretase activity on APP processing, while in endosomes, it facilitates BACE1‐mediated APP cleavage [30, 43]. Evidence also suggests the presence of BACE1 in the TGN, supporting the idea that Aβ generation occurs within neurons during the early stages of AD [44, 45].

Moreover, lipid rafts play a crucial role in determining the mechanisms and locations of APP processing. The amyloidogenic processing of APP is facilitated by the activity of BACE1 and γ‐secretase within lipid rafts. Notably, reducing cholesterol levels in N2a neuroblastoma cells has been shown to decrease Aβ production [46, 47]. Given this evidence, the modulation of lipid metabolism stands out as a compelling new paradigm for understanding and treating AD [48]. We will further explain the interaction of Aβ with lipid rafts and its involvement in Aβ pathogenesis in Section 6.

### 3.3 Physiologic Functions of APP and Its Derivatives

The precise physiological functions of APP remain to be completely understood. APP was initially identified as a cell surface receptor and a synaptic adhesion molecule [30]. The extracellular domain of APP interacts with extracellular matrix proteins, including transient axonal glycoprotein‐1 (TAG1), Reelin and F‐spondin [49, 50, 51, 52]. Transmembrane APP family members can form homo‐ and hetero‐ cis‐ or trans‐ dimerization, enabling diverse physiological functions. The major physiological functions of APP include its role as an adhesion molecule at the neuromuscular junction and its involvement in various cellular processes such as cell growth, motility, neurite outgrowth, neural migration, synaptogenesis, synaptic plasticity, and promotion of cell survival. These functions have been experimentally established both in vitro and in vivo [30, 32, 33, 53, 54, 55]. Many of these functions are believed to be mediated by sAPPα, which is generated during the non‐amyloidogenic processing of transmembrane APP. Furthermore, the injection of interfering RNA targeting the *APP* gene (APP‐RNAi) into rodent fetuses has provided evidence of aberrant neuronal migration, further supporting the role of APP in regulating normal neuronal distribution [54]. Further, neuronal outgrowth may be regulated by APP through its interaction with extracellular proteins such as Reelin and heparan sulfate proteoglycan. The distribution of the phosphorylated version of mature APP at the Thr668 residue in the growth cone during neuronal differentiation in PC12 cells suggests a possible role for APP in neuronal path finding during development [50, 56, 57]. Studies have also demonstrated that intracranial injection of sAPPα improves cognitive abilities and increases synaptic density in adult animals, suggesting a role for APP in memory formation [33]. Furthermore, the age‐related decline in neural progenitor cell proliferation can be reversed by injecting recombinant sAPPα into the ventricles, highlighting its role in brain plasticity [58].

APP has an interplay with three major neurotransmitter systems including glutamatergic, cholinergic, and GABAergic that are known to be altered in AD [59, 60, 61]. While APP increases GluN2B‐containing N‐methyl‐D‐aspartate (NMDA) receptor levels and function in the membrane, NMDA receptor stimulation, in turn, reduces the surface expression of APP and promotes amyloidogenesis [62]. GluN2B is frequently associated with excitotoxicity and neuronal degeneration in the adult brain, whereas GluN2A is linked to plasticity and pro‐survival pathways [63]. Furthermore, APP regulates the equilibrium of D‐serine, a potent natural co‐agonist of the NMDA receptor. In support of this argument, there is evidence that knock out mice lacking APP exhibit defects in the structural flexibility of dendritic spines that are dependent on D‐serine [64].

APP undertakes a significant role in cholinergic signal transmission at the neuromuscular junction and throughout the autonomic nervous system [65, 66]. It has been suggested that sAPPα binds to α7‐type nicotinic acetylcholine receptors (α7‐nAChRs), acting as an endogenous positive allosteric modulator of cholinergic signaling [67]. The APP protein is abundantly expressed in GABAergic interneurons and plays a crucial role in modulating both phasic and tonic inhibitory functions. APP inhibits the L‐type calcium channel CaV1.2, which, in turn, inhibits tetanic potentiation in striatal and hippocampal GABAergic interneurons. This remark suggests that APP regulates synaptic properties in GABAergic neurons through the control of CaV1.2 channels [55, 68]. Furthermore, sAPPα is crucial for maintaining intracellular calcium balance and facilitating synaptic transmission by modulating NMDA and GABA receptors [32, 69].

As one of the main derivative products of APP cleavage and the most relevant to AD, the Aβ peptide serves physiological functions and plays a crucial role in synaptic physiology by regulating synaptic function scaling and the release of synaptic vesicles [30, 33]. Additionally, Aβ has been suggested to have several potential functions, including stimulation of kinase enzymes [70, 71], protection against oxidative stress [72, 73], regulation of cholesterol transport [74], involvement in calcium homeostasis and electrical activity of the brain [75], as well as antimicrobial activity [76]. Aβ also contains the exact amino acid sequence needed for the interaction between sAPPα and α7‐nAChRs. Because of this, it is not surprising that Aβ has a strong binding affinity for these receptors at both presynaptic and postsynaptic sites [77].

The deletion of APP in adult mice, which eliminates Aβ production, does not appear to significantly impact their phenotype. This suggests that neither APP nor Aβ is essential for normal physiological function in adulthood. However, data indicate that triple knockout of APP, APLP1, and APLP2a leads to dispersed aberrant migration of cortical neurons. Furthermore, mismatching of presynaptic and postsynaptic markers at the neuromuscular junction and excessive nerve terminal outgrowth are observed in mice with double knockouts of APP and APLP2 [33]. To further investigate the role of APP in neurodegenerative disorders, numerous mouse models have been developed, incorporating modified expression or mutant variants of APP [78, 79]. Nevertheless, the precise physiological function of APP in the adult CNS remains to be understood and requires further research.

Two roles have been hypothesized for the intracellular C‐terminal of APP, which are crucial for its functionality. One function is to regulate transcription, while the other is associated with the YENPTY domain, a highly conserved sequence from flies to humans, functioning as an intracellular regulator of APP [30, 33]. The YENPTY domain serves as a structure for protein sorting, with adaptor proteins like X11 and Fe65 exhibiting binding affinity toward it. The adaptor proteins regulate the internalization of clathrin‐coated pits by binding to the YENPTY domain [80]. Mutations in the YENPTY site alter the APP endocytosis and decrease Aβ synthesis. The adaptor proteins X11 and Fe65, which exhibit significant expression levels within the brain, engage in interactions with all members of the APP family. The association of APP with SorLA/LR11 at the trans‐Golgi network (TGN) is facilitated by these adaptor proteins, hence inhibiting the interaction between APP and BACE1 [33, 81]. The SorLA/LR11 receptor, also referred to as LR11, is a type‐1 receptor with a molecular weight of 250 kD. This nomenclature is ascribed to its structural similarity to the conventional constituents of the low‐density lipoprotein (LDL) receptor family. The protein exhibits a high level of expression in several brain regions, including the cerebral cortex, hippocampus, and cerebellum as well as the spinal cord [82]. The overexpression of X11 and Fe65 in mice results in a reduction in the Aβ accumulation [80, 83]. Nevertheless, in AD, there is a notable reduction in the levels of X11, Fe65, and SorLA/LR11 [33, 84]. The retrograde trafficking of endosomal APP into the TGN is regulated by SorLA/LR11, which exerts its influence via interacting with retromer molecules. These findings highlight the antagonistic effect of SorLA/LR11 on Aβ production in AD [85, 86].

## 4 Amyloid β (Aβ)

### 4.1 Aβ Species and Their Neurotoxicity in Pathogenesis of AD

**FIGURE 3:**
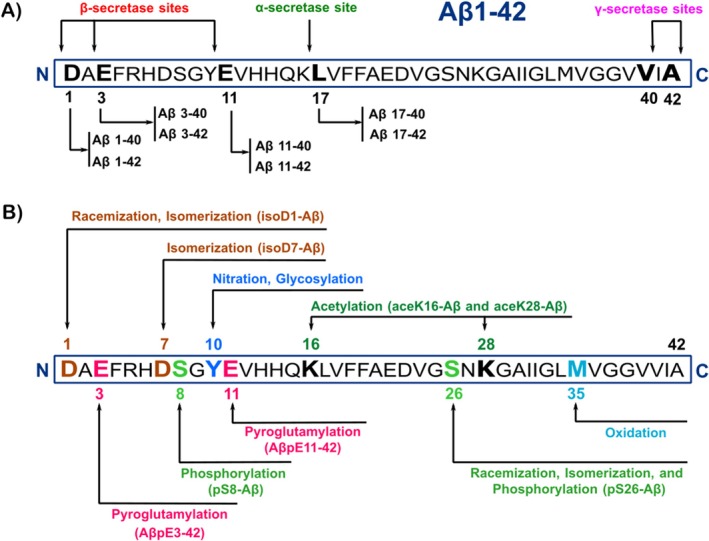
Aβ species of varying lengths and with different post‐translational modifications. (A) The sites of action for different secretases on Aβ1‐42 and the Aβ species they produce. (B) Post‐translational modifications of Aβ at specific residues, which result in isoforms with different toxicities. Isomerization of aspartate at residues 1 or 7 produces isoD1‐Aβ or isoD7‐Aβ. Pyroglutamate formation at glutamate residues 3 and 11 results in AβpE3‐42 and AβpE11‐42. Phosphorylation of serine at residues 8 and 26 produces pS8‐Aβ and pS26‐Aβ. Acetylation of lysine at residues 16 and 28 results in aceK16‐Aβ and aceK28‐Aβ. Amino acid residue abbreviations: D, Aspartic acid; E, Glutamic acid; S, Serine; Y, Tyrosine; K, Lysine; M, Methionine. The letters N and C at the ends of the Aβ peptide represent the N‐ and C‐termini, respectively.

While a growing body of evidence from experimental and clinical studies has challenged the prominent role of Aβ in AD development and progression [87, 88], the classical amyloid cascade hypothesis postulates the accumulation of Aβ as the initiating cause of the disease [89, 90]. The heterogeneity of Aβ species is thought to underlie the distinct phenotypic presentations of AD [91, 92]. Aβ peptides of varying lengths result from either the primary enzymatic cleavage of APP or the secondary processing of Aβ by exopeptidases [93]. BACE1 cleaves APP at specific sites to initiate amyloidogenic processing. Subsequently, γ‐secretase cleaves APP at various locations, generating a spectrum of Aβ species ranging from 34 to 43 amino acids in length, such as Aβ1‐40, Aβ1‐42, and Aβ1‐43 (Figure 3A). Among these Aβ species, Aβ1‐42 ending with the alanine at position 42 has a stronger tendency to aggregate as compared with Aβ1‐40. These species are thought to be the driving factor for the formation of amyloid plaques and the neurotoxic effects [94]. The main component of amyloid plaques and CAA in AD is fibrils, which measure 10–20 nm in width and can extend beyond 1 μm in length. The fibrils are made up of peptide strands linked by intermolecular β‐sheets. The cross‐β structure is created when these β‐strands, which are perpendicular to the main fibril axis, link together parallel by hydrogen bonds [95].

Furthermore, several N‐ and C‐terminally truncated or modified forms of Aβ peptide species, which play a key role in the pathogenesis and progression of AD, are also found in the patient brains [93, 96]. For instance, the imprecise cleavage site of γ‐secretase at the C terminal of the Aβ domain produces even shorter Aβ isoforms such as Aβ1–17, Aβ1‐18, Aβ1‐19, and Aβ1‐20 [97]. In addition, N‐terminally truncated Aβ peptides, especially the Aβx‐42 fragments such as Aβ3‐42 and Aβ11‐42, are the toxic species probably involved in the initial step of Aβ accumulation and AD development. Valverde et al. [98] identified aminopeptidase A as the primary exopeptidase responsible for the N‐terminal truncation of Aβ in a mouse model of AD. Furthermore, they demonstrated that pharmacological inhibition of this enzyme rescued memory impairments, supporting the role of the Aβ3‐42 fragment in AD‐related cognitive deficits. A successful AD therapy would need to detect these species early, before clinical symptoms and pathology are evident, and block the harmful biochemical cascades they trigger [99].

### 4.2 Role of Post‐Translational Modifications of Aβ in AD Pathogenesis

It has been indicated that the pathogenesis of AD and the distinct disease phenotypes is driven not only by the abundance of a variety of Aβ peptides but also by their specific biochemical properties, which are profoundly influenced by post‐translational modifications (PTMs) [100]. PTMs have recently emerged as hidden players in the pathogenesis of neurodegenerative diseases [101, 102]. According to research, the incorporation of the modified Aβ species into aggregates enhances their resistance to proteolytic breakdown, reducing Aβ clearance and increasing its concentration and toxicity [94]. Aβ can interact non‐covalently with metal ions like zinc, as well as with lipids, proteins, and other peptides [103, 104]. These interactions have significant impacts on the aggregation tendency of Aβ. For instance, research indicates that abnormal Aβ‐metal ion interactions contribute to the onset of sporadic AD [104]. Growing evidence also indicates that PTMs significantly influence the Aβ aggregation process, promoting the formation of toxic species [97, 105]. The Aβ PTMs include oxidation, nitration, racemization, isomerization, pyroglutamylation, phosphorylation, acetylation, and glycosylation (Figure 3B).

Some of the PTMs, such as oxidation and nitration, are clearly induced by oxidative stress and neuroinflammation [97]. Pyroglutamylation mainly occurs at 3 and 11 glutamate residues, which include removal of N‐terminal residues and cyclization to form two types of pyroglutamates Aβ species (AβpE3‐42 and AβpE11‐42). Research indicates that these Aβ species have more hydrophobicity and resistance to degradation [106]. These characteristics cause the construction of highly stable, oligomeric, and neurotoxic aggregates that seed further plaque formation. Additionally, it has been shown that AβpE3‐42 is involved in glial release of the proinflammatory cytokine TNFα and the onset of synaptic dysfunction in AD [107].

Research indicates that Aβ phosphorylation at serine residues 8 and 26 (pS8‐Aβ and pS26‐Aβ, respectively) by extracellular or cell surface‐localized protein kinase A enhances Aβ stability and its rate of aggregation [100, 108]. Kumar et al. [108] demonstrated that pS8‐Aβ is resistant to proteolytic degradation by insulin‐degrading enzyme, thereby reducing Aβ clearance by microglial cells. Studies have proposed that the modifications including isomerized Asp7 and phosphorylated Ser8 (isoD7‐pS8‐Aβ) could protect the Aβ peptide from zinc‐induced oligomerization in mice [109]. Phosphorylated Aβ pS8‐Aβ has been detected in plaques and contributes to neuronal dysfunction by increasing oligomer toxicity and promoting Tau hyperphosphorylation. Therefore, it may form a pathogenic link between Aβ and Tau pathology in AD [108]. pS26‐Aβ assembles into a specific oligomeric form that does not proceed further into larger fibrillar aggregates and plaques but increases Aβ oligomer toxicity in human neurons [100].

Racemization and isomerization are age‐dependent modifications that occur at aspartate residues 1 and 7 of Aβ. Racemization converts L‐aspartate to its D‐isomer, while isomerization forms isoaspartate. These modifications, particularly at Asp7, alter the structure and morphology of Aβ, causing it to exhibit increased neurotoxicity and induce amyloidogenesis in the brain tissue of transgenic mice [110]. Furthermore, Barykin et al. [111] demonstrated that isomerization at Asp7 (isoD7‐Aβ) enhances Aβ neurotoxicity by inhibiting the function of the α7‐nAChR, which disrupts the cholinergic system in AD. Other investigators have also reported that acetylation of Aβ at lysine 16 and 18 residues (aceK16‐Aβ and aceK28‐Aβ, respectively), or at both, significantly increases oxidative stress, ROS production, and inhibits the proliferation of SH‐SY5Y cells [112].

Elevated levels of special species of Aβ may also play a role in the transition from pre‐AD to symptomatic AD [95]. Both pre‐clinical and symptomatic phases exhibit the presence of unmodified Aβ42 as well as modified versions [95]. Certain data indicate the absence of AβpE3‐42 during the pre‐clinical phase. However, in contrast to pSer8Aβ and AβpE3‐42, AβpE11‐42 shows a stronger association with symptomatic stages of AD [95, 113]. The process of maturation of Aβ, which involves heightened concentration, PTMs, and an increase in insoluble aggregates, is observed in conjunction with the transition from the neocortex to other brain regions, as well as the transformation from pre‐clinical AD to symptomatic AD [95]. Therefore, it can be proposed that PTMs of Aβ play a pivotal role in the spread of the disease to other brain regions and in the severity of AD.

Despite advances in identifying oligomeric conformations, questions remain regarding the relationship between structural characteristics and biological functions, as well as the relative toxicity of different oligomeric species in neurons [114]. β‐sheet interfering molecules effectively inhibit Aβ aggregation and fibril formation [115]. Nevertheless, the neurotoxic effects of Aβ plaques remain incompletely understood, as the underlying processes responsible for their toxicity have not been thoroughly elucidated [89]. Research reveals that the binding of Aβ to the neuronal membrane occurs through the attraction with the sugar group of GM1 ganglioside, a glycosphingolipid abundant in lipid rafts. This interaction diminishes the predisposition of Aβ to adopt a β‐sheet shape and engage in oligomerization. Hence, GM1 ganglioside has the potential to serve as a protective factor against toxicity associated with Aβ aggregation [116]. These findings underscore the significance of alterations in Aβ species, encompassing interactions with metal ions and other biomolecules in addition to PTMs. These modifications contribute significantly to pathogenesis while also modulating cerebral amyloidosis and the ensuing plaque formation characteristic of AD. However, unraveling the intricate network of Aβ PTMs and their precise impact on disease mechanisms remains a crucial goal for future studies.

### 4.3 Anatomical Distribution of Aβ in Different Brain Areas in AD

The distribution of extracellular monomeric Aβ and soluble aggregates across various brain areas is facilitated through passive and/or active transport mechanisms. The interconnections between brain regions may further promote the spread of Aβ [117]. In addition to neurons, intracellular Aβ has been observed in microglia and astrocytes, contributing to the distribution of Aβ in the brain [118, 119]. Both Thal and Braak have independently identified distinct stages of Aβ plaque deposition, which serve as pathological markers for AD progression.

**FIGURE 4:**
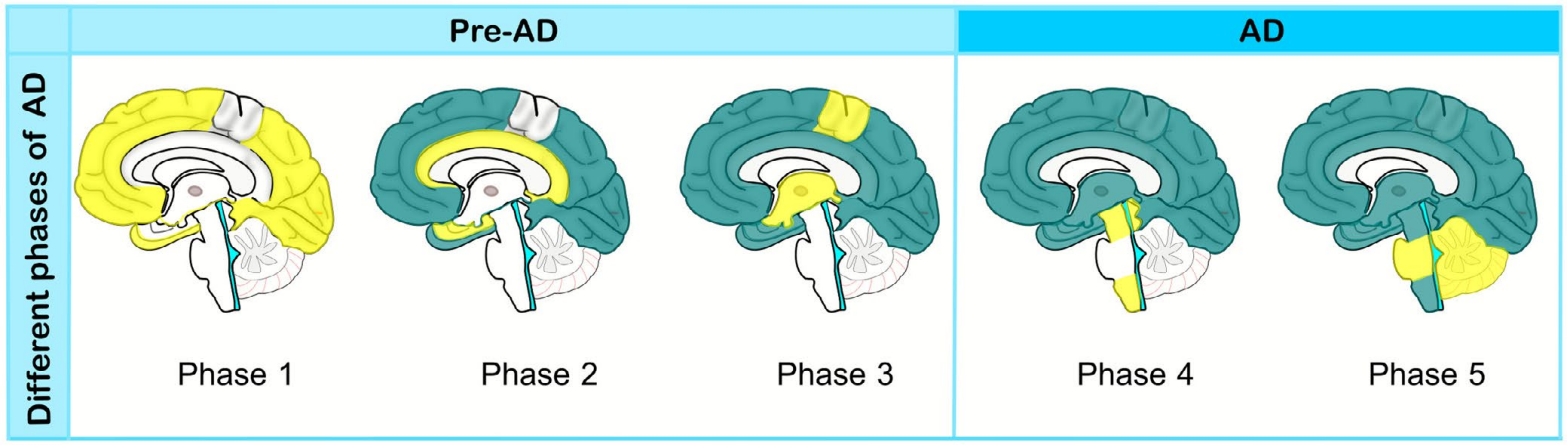
The progression of Aβ plaques in the AD brain. Each brain figure illustrates the distribution of Aβ plaques across different brain regions according to Thal's five‐phase model of amyloid pathology in pre‐AD and AD stages. In each phase, newly affected areas are marked in yellow, while previously involved regions are shown in dark blue. White areas are still intact in each phase.

Thal's five‐phase model suggests that amyloid plaques initially accumulate in the neocortex before spreading to allocortical regions, including the entorhinal cortex, hippocampus, and cingulate gyrus. By phase 3, plaques appear in the striatum, hippocampus, thalamus, and basal forebrain. Phase 4 involves the midbrain and medulla oblongata, followed by phase 5, where plaques extend into the cerebellum and pons [95, 120]. Similarly, Aβ plaque accumulation begins in the cortical vasculature and leptomeningeal region, then spreads to the allocortical and cerebellum during phase 2. The ultimately affected structures include the basal ganglia, diencephalon, brainstem, and white matter (Figure 4) [95]. According to Braak's stages, Aβ plaques first appear in the basal frontal and temporal lobes before extending to the neocortices and hippocampus. Eventually, they reach the primary cortex, subcortical nuclei, and cerebellum [121]. However, further research is needed to understand the contributions of distinct Aβ species within different brain regions, their inter‐regional spread, and their relationship to the varied pathological presentations of AD.

### 4.4 Mechanisms Underlying Age‐Related Aβ Accumulation

The accumulation of extracellular Aβ in the brain is attributed to an imbalance between its production and clearance [3, 16, 23]. APP undergoes continuous metabolism in the brain, resulting in the formation of Aβ, which is subsequently transported into cerebrospinal fluid (CSF) for clearance. However, in the early stages of AD, there is a decline in the concentration of Aβ in the CSF, while it is elevated in the brain tissues indicating impaired transfer of Aβ and its clearance from the brain tissue into the CSF.

Several factors contribute to Aβ accumulation in the brain, including an altered Aβ42/Aβ40 ratio, impaired CSF clearance, and the presence of chaperone molecules such as APOE and clusterin, which influence Aβ aggregation and deposition [33]. In addition to LRP1‐mediated Aβ clearance, the extracellular elimination of Aβ is facilitated by neprilysin, insulin degrading enzyme, polyfunctional endothelial transporter proteins P‐glycoprotein, and receptor for advanced glycation end products (RAGE). Consequently, modifying any of these proteins decreases Aβ clearance, resulting in Aβ accumulation in the brain [23, 33].

The accumulation of Aβ in the brain can also occur through a third pathway involving altered APP proteolytic processing. This includes: (1) mutations in APP that lead to FAD by shifting cleavage toward amyloidogenic pathways, and (2) alternative processing routes that generate heterogeneous Aβ species with enhanced aggregation propensity [6, 33]. Acetylation of the α‐secretase gene can suppress its enzymatic activity, thereby inhibiting the non‐amyloidogenic pathway. This epigenetic modification simultaneously enhances β‐secretase activity, promoting the amyloidogenic pathway and ultimately accelerating Aβ accumulation [33, 89].

### 4.5 Aβ and Oxidative Stress: Partners in AD

The primary cause of oxidative stress is the production of free radicals during mitochondrial respiration [122]. Mitochondrial dysfunction is associated with increased production of reactive oxygen species (ROS), disrupted intracellular calcium levels, and decreased ATP production [123]. Research from the Reddy Laboratory and others shows that altered mitochondrial dynamics—specifically, increased fission and decreased fusion—may be a primary cause of both mitochondrial dysfunction and subsequent neuronal damage. They reported that an increase in dynamin‐related protein 1 (Drp1) causes mitochondrial fragmentation via activation of the mitochondrial fission machinery, which in turn leads to mitochondrial dysfunction and neuronal damage [124, 125]. This concept is further supported by evidence that Drp1 can interact directly with both Aβ and Tau protein in human brain tissues and mouse models of AD [126]. Furthermore, Aβ triggers excessive nitric oxide (NO) production, which mechanistically promotes mitochondrial fission in neurons via S‐nitrosylation of Drp1, ultimately resulting in synaptic degeneration and neuronal damage [126].

Furthermore, mitochondrial membranes contain protein import channels that are crucial for the transport of nuclear‐encoded proteins into the organelle. Research indicates that in human AD brains, APP accumulates specifically within these channels, inhibiting this essential transport process and thereby disrupting mitochondrial function and cellular homeostasis [127]. The presence of Aβ within mitochondria has been consistently documented in various models, such as cell cultures, transgenic mice, and postmortem human brain tissue [128], although the mechanism by which mitochondrial Aβ is produced is not clear [127, 129]. While both a γ‐secretase‐like protease and APP are present in mitochondria, the specific orientation of APP on the mitochondrial membrane makes it unlikely that Aβ is produced inside the organelle. A more plausible explanation is that Aβ is generated externally and then imported into the mitochondria [127]. Research indicates that Aβ has an inhibitory effect on integrated mitochondrial respiration as well as on the activity of key enzymatic components. Within the mitochondria of AD patients and transgenic mice, Aβ binds to a vital energy regulator called alcohol dehydrogenase to form the complex Aβ‐binding alcohol dehydrogenase (ABAD). This binding induces a conformational change in ABAD's active site, thereby preventing the binding of nicotinamide adenine dinucleotide (NAD) [130]. Aβ in the mitochondria also inhibits cytochrome oxidase activity and elevates ROS [123]. Ultimately, these changes in the mitochondria create a vicious cycle: increased ROS drives further Aβ production, which in turn exacerbates mitochondrial dysfunction and leads to neuronal damage [131].

Oxidative stress has been observed to impact mRNA, tRNA, and rRNA, resulting in a decrease or cessation of protein synthesis in both the mitochondria and cytoplasm [132]. Furthermore, oxidative stress affects the expression of several miRNAs in susceptible brain regions of patients with AD. In addition, oxidized miRNAs lead to the misidentification of target mRNAs [133]. Together, Aβ in the mitochondria mediates neurotoxicity through multiple interconnected mechanisms, including the induction of mitochondrial dysfunction, generation of reactive oxygen species (ROS), activation of apoptotic pathways, and finally disruption of synaptic integrity and function [117, 118]. However, how exactly Aβ and other AD‐associated factors lead to mitochondrial dysfunction and neuronal damage remains an open question and warrants further investigation.

The processes of mitochondrial dynamics have pivotal roles in preserving mitochondrial functions in the cells. Additionally, research indicates that mitophagy, a selective form of autophagy, acts as a clean‐up system by removing defective mitochondria, thereby reducing the free radical burden [134]. Mitophagy enhancers such as urolithin A—a natural compound—may boost the clearance of Aβ‐damaged mitochondria by enhancing lysosomal function [135, 136]. As previously mentioned, Aβ accumulation and mitochondrial dysfunction have a bidirectional relationship [137]. Mitophagy enhancers can break this cycle by restoring mitochondrial and synaptic health. This reduces ROS levels, which in turn decreases both Aβ and hyperphosphorylated Tau, along with their associated toxicities [134, 135]. Although significant support exists for mitophagy enhancers as promising AD therapeutics, they require careful testing in mouse models with late‐onset AD features before advancing to clinical trials [135].

### 4.6 Aβ Induces Neuroinflammation

**FIGURE 5:**
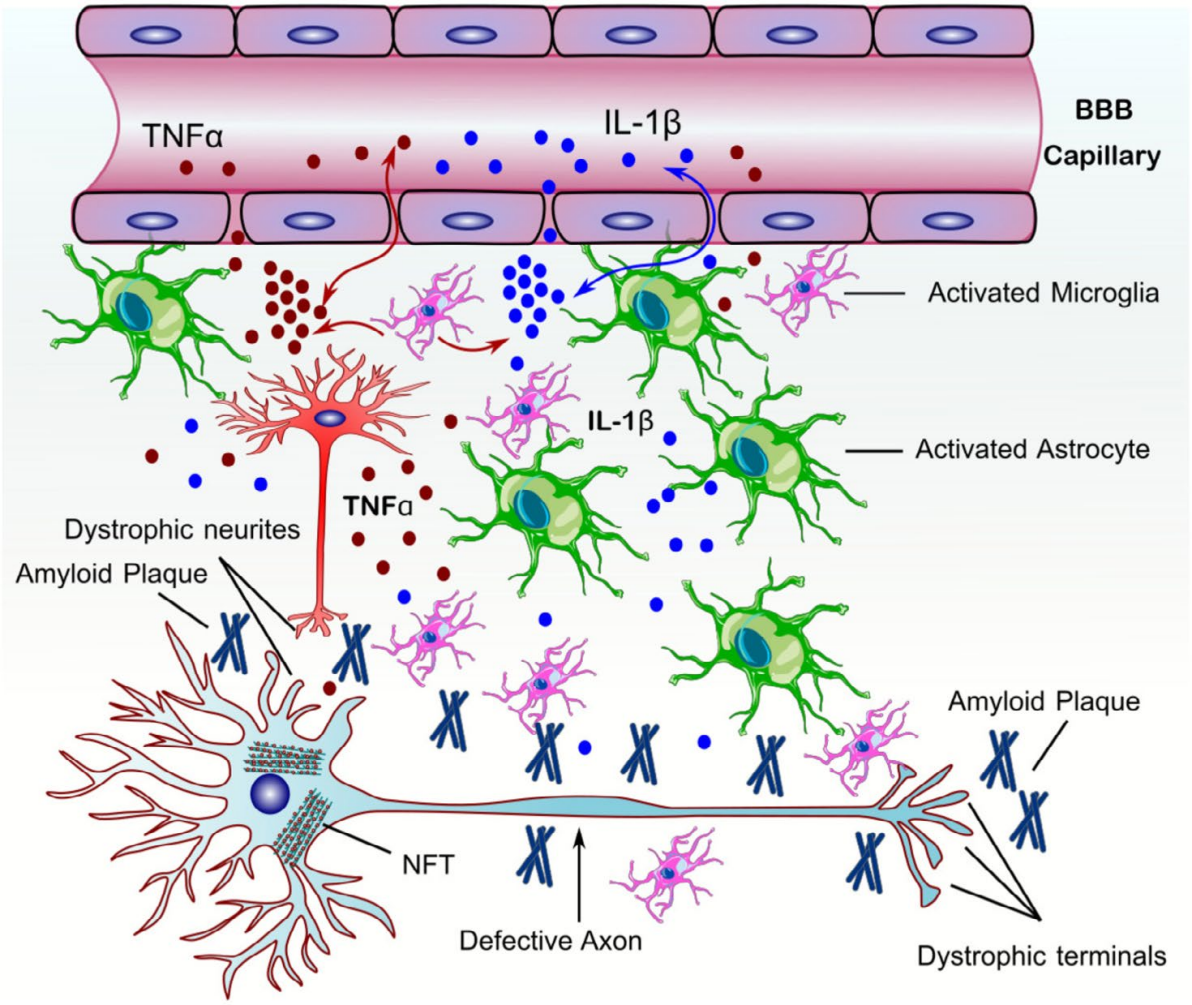
Aβ‐induced neuroinflammation and glial activation. The aggregation of Aβ leads to the activation of microglia and recruitment of astrocytes. This process leads to the release of cytokines and chemokines, which have detrimental effects on neurons. Abbreviations: IL‐1β, Interleukin 1β; TNFα, Tumor necrosis factor α; BBB, Blood–brain barrier; NFT, Neurofibrillary tangle.

Neuroinflammation is a crucial factor in the initiation and progression of neuropathological changes observed in AD. The significance of inflammation is underscored as one of the important pathological elements in the pathophysiology of AD [138]. Research suggests that Aβ fibrils trigger inflammatory responses in microglia, leading to neuronal death [138]. Microglia play a role in inflammation, which can influence the progression of AD. Microglial cells respond to Aβ through two key functions. First, they facilitate Aβ uptake by engulfing and internalizing it. Second, they contribute to Aβ degradation by releasing enzymes such as IDE, which break down Aβ and reduce its toxicity [139]. The persistent immunological response generated by Aβ is ascribed to the activation of microglial cells and the heightened secretion of cytokines and chemokines (Figure 5). Microglia and immune cells have been shown to increase both Aβ and Tau pathology [140, 141]. The temporal relationship between neuroinflammation and Aβ pathology remains unresolved: it is unclear whether proinflammatory cytokines precede and initiate Aβ deposition, or whether Aβ accumulation itself triggers subsequent immune activation.

In contrast, microglia have been shown to play a crucial role in the elimination of Aβ by releasing several proinflammatory cytokines and toxic substances, including ROS and NO [142]. The synthesis of these compounds facilitates the recruitment of more microglia (microgliosis) and astrocytes (astrocytosis) in close proximity to Aβ plaques. The stimulation of the NF‐κB pathway in astrocytes by inflammatory agents, such as Aβ, can lead to an augmented release of C3 complement. This, in turn, interacts with the C3α receptor on microglia and neurons, ultimately resulting in the activation of microglia and subsequent neuronal dysfunction [143]. Besides, Aβ can also be up taken by microglia, resulting in the activation of the NF‐κB and the mitogen‐activated protein kinase (MAPK) signaling pathway that in turn induces proinflammatory gene expression [30, 144]. The aforementioned data provide evidence that persons with AD exhibit an upregulation of inflammatory cytokines and chemokines, including IL6, IL8, TNFα, TGF‐β, and macrophage inflammatory protein‐1γ, in comparison to control individuals [138].

Astrocytes, in their reaction to persistent inflammation, may also secrete β‐secretase, leading to the production of further Aβ. Increased expression of several surface receptors, including major histocompatibility complex (MHC) and complement, is observed in activated microglia [145]. The dynamic interplay between microglia and astrocytes causes a reciprocal feedback mechanism, leading to the amplification of the inflammatory response [146]. Another possible source of astrocytosis and neuronal death is the C‐terminal portion of the APP molecule, which is seen in senile plaques [16]. Moreover, it has been observed that animal mutant models that exhibit overexpression of APP or Tau protein demonstrate heightened immunoreactivity. Hence, targeting inflammation induced by Aβ, or the underlying causes of Aβ accumulation, can be considered a potential therapeutic objective in AD.

According to epidemiological studies, individuals who have been using non‐steroidal anti‐inflammatory drugs (NSAIDs) for an extended period of time exhibit a reduced susceptibility of up to 50% in developing AD [138, 147]. The protein adiponectin, produced by adipocytes, inhibits the inflammatory response of microglia to Aβ oligomers via modulating the adipoR1/AMP‐activated protein kinase (AMPK)/NF‐κB signaling pathway. This indicates that adiponectin may be a potential therapeutic factor for neuroinflammation caused by Aβ in AD [148].

Triggering receptor expressed on myeloid cells 2 (TREM2), which is expressed predominantly on microglia, was found to influence the AD. Previous studies demonstrated the protective role of TREM2 in response to amyloid pathology [149]. Wang et al. [149] demonstrated in the 5XFAD mouse model of AD that microglia failed to fully engulf Aβ plaques when TREM2 was absent. The researchers proposed that TREM2 plays a protective role in AD by allowing microglia to surround and remodel Aβ plaques, thereby reducing neuritic damage. Together, it can be concluded that TREM2 significantly influences how the innate immune system responds to Aβ pathology.

### 4.7 Role of Endolysosomal‐Autophagic Malfunction in Aβ Accumulation

Endolysosomal trafficking and autophagy play crucial roles in the pathological mechanisms of AD [150]. Under normal conditions, lysosomes degrade misfolded proteins and damaged organelles through the macroautophagy route. However, lysosomal dysfunction, which begins with aging, can result in Aβ accumulation [151]. Since autophagy plays a role in clearing intracellular Aβ, impaired lysosomal function hinders Aβ clearance, promoting Aβ oligomerization and increasing toxicity [150, 152]. Moreover, disruptions in the lysosomal and autophagy pathways lead to the buildup of defective organelles, such as mitochondria causing the release of harmful substances that contribute to cellular death and the accumulation of autophagic vacuoles. Growing evidence suggests that lysosomal deficiencies during autophagy are associated with numerous AD features, such as dystrophic neurites, apoptosis, and Tau pathology, in addition to Aβ accumulation [4, 153]. Furthermore, during the initial phases of AD, the accumulation of insoluble Aβ can cause the destruction of endosome and lysosome membranes, leading to the disruption of their normal functioning [154]. Autophagic vacuoles play a defensive function in protecting against Aβ. When the toxicity load of Aβ increases, it leads to a decrease in the breakdown and release of lysosomal enzymes from abnormal autophagic vacuoles, which in turn harms neurons [4, 15]. γ‐secretase complexes are highly concentrated in endocytic and autophagic vacuoles. Therefore, any disruption in the autophagic pathway has been consistently shown to contribute to the production of Aβ and the development of AD [15].

PS1 and PS2 serve as catalytic components of the γ‐secretase complex, as stated earlier. They play a crucial role in the breakdown of type 1 transmembrane proteins, particularly APP. Additionally, they are involved in cell signaling, maintaining calcium balance, intracellular trafficking within endosomes and lysosomes, and the process of autophagy [155]. PS1 acts as a chaperone molecule in the endoplasmic reticulum, facilitating the maturation of the proton pump (v‐ATPase) found in endosomes and lysosomes. This pump is crucial for maintaining proper acidification and pH regulation within these organelles [43]. Mutations in the PS enzyme can cause the progression of AD by disrupting the function of endosomes and lysosomes, regardless of its proteolytic role in APP processing and the development of FAD [22, 43]. In addition, a deficiency in the PS enzyme leads to an elevation in intracellular calcium levels due to an increased release of calcium from the endoplasmic reticulum and lysosome. This, in turn, activates calcium‐dependent enzymes such as Calpain [156]. The target molecules of Calpain encompass cytoskeletal proteins, signaling proteins, and transcription factors. Calpain exerts its neurotoxicity by enhancing the activity of cyclin‐dependent kinase 5 (CDK5), extracellular signal‐regulated kinase 1,2 (ERK1,2), and glycogen synthase kinase‐3β (GSK3β). Calpain can also induce aberrant phosphorylation of Tau protein and the formation of NFTs by altering intracellular signaling pathways [153]. Thus, mutations in PS1, regardless of the proteolytic processing of APP, can lead to FAD by impairing lysosome function, lowering Aβ clearance, and increasing calpain activity [4, 15]. Studies have shown that peroxisome proliferator activated receptor alpha (PPARA/PPARα) regulates autophagy within the nervous system and PPARA‐mediated autophagy may influence AD. Notably, PPARA agonists stimulate autophagy in human microglia and U251 human glioma cells leading to a reduction in Aβ pathology and the reversal of memory impairment and anxiety symptoms in APP‐PSEN1E9 mice [157].

### 4.8 Aβ Prevents Neurogenesis in AD

Neurogenesis in the CNS of healthy adults occurs throughout life in certain brain regions, including the olfactory bulb, subventricular zone, and hippocampal dentate gyrus [158]. Hippocampal neurogenesis has been well documented to decline with age in animal models and in patients with mild cognitive impairment (MCI) and AD [159, 160, 161]. However, in contrast to the findings of Sorrells et al. [162], studies suggest that human hippocampal neurogenesis persists in aged adults and AD patients [163]. A decline in the number of neuroblasts has been observed in MCI and AD patients. Accumulating evidence indicates that Aβ promotes neuronal atrophy in AD through the induction of necroptosis and ferroptosis [164]. A higher number of neuroblasts and greater functional interaction between SNARE proteins, syntaxin and SNAP‐25, is associated with cognitive function. Conversely, lower levels of presynaptic proteins correlate with cortical atrophy and cognitive impairments in AD [163, 165].

The neurogenesis process has been shown to be accompanied by an increase in cyclin‐dependent kinase 5 (CDK5) and its two activators, p35 and p25 [166]. CDK5 is the predominant CDK in the brain, abundantly expressed in neurons, and plays a significant role in synaptic plasticity and neuronal development [167]. CDK5 is a serine–threonine protein kinase with post‐mitotic activity that phosphorylates cell skeletal proteins (Tau, neurofilament, and nestin), synaptic proteins (synapsin, cadherin and PSD95), and transcriptional factors (MEF2) [168]. Unlike other CDKs, which are activated by cyclins in dividing neurons, CDK5 is activated by forming a complex with p35 or p39. However, p35 is the primary activator of CDK5, regulating its physiologic function [169]. CDK5/P35 is involved in neuroblast migration and synaptic plasticity. Moreover, in axonal outgrowth cones, this complex regulates neurotic outgrowth in mature cortical neurons. Therefore, it plays an essential role in adult neurogenesis. However, Aβ‐induced calcium influx activates calpain, a protease that cleaves p35 into p25. The resulting P25 hyperactivates CDK5, leading to the abnormal phosphorylation of substrates such as Tau [169, 170]. This implies that Aβ disrupts neurogenesis via activating calpain protease and reducing p35 expression. These findings indicate that impaired neurogenesis in AD is linked to the dysregulation of CDK5 in neural progenitor cells [23]. The mechanistic interplay between Aβ pathology and impaired neurogenesis in the AD brain, however, requires further elucidation.

## 5 Role of APOE in AD

APOE, a member of the apolipoprotein family, is encoded by the *APOE* gene located on chromosome 19 in humans. The *APOE* gene has three variants, including *APOE ε2*, which is rare in the population, *APOE ε3*, which is the most prevalent allele, and *APOE ε4* [33]. The differential distribution of APOE isoforms across lipoprotein particles results from a single amino acid substitution—arginine (Arg) to cysteine (Cys)—at two key residues: position 112 (Cys in APOE2 and E3, Arg in APOE4) and position 158 (Cys in APOE2, Arg in APOE3 and E4). In the brain, APOE4 is the primary apolipoprotein associated with chylomicron and very low‐density lipoprotein (VLDL) metabolism. In contrast, APOE2 and APOE3 are primarily associated with high‐density lipoprotein (HDL) [171]. APOE is expressed in both peripheral organs and the CNS, with the liver being the major source of peripheral APOE. Within the CNS, APOE is predominantly produced by astrocytes and vascular cells, with additional contributions from choroid plexus cells. Under pathological conditions, activated microglia and stressed neurons may also synthesize APOE, albeit to a lesser extent. Due to the blood–brain barrier (BBB), APOE in the CNS and peripheral organs forms distinct pools. Within the CNS, APOE functions as a key lipid transporter, delivering cholesterol and other lipids to neurons through binding with APOE receptors—particularly LDL receptor‐related protein 1 (LRP1), which is highly expressed on neuronal surfaces [171, 172]. The APOE ε4 variant is associated with elevated cholesterol levels and increased risk of arteriosclerosis, a condition linked to both hypercholesterolemia and AD. This evidence has led to the hypothesis that dysregulated cholesterol metabolism may contribute to AD pathogenesis [173]. Cholesterol and lipids transported by APOE play crucial roles in synaptogenesis, with these functions being modulated by isoform‐specific effects. Notably, APOE3 (but not APOE4) promotes neurite outgrowth and enhances tissue repair following neural injury [171]. Beyond its physiological roles in the brain, APOE contributes to AD pathogenesis through its interaction with Aβ. APOE co‐aggregates with Aβ in plaques, accelerating both Aβ deposition and fibril formation. This process correlates with a decreased Aβ42/40 ratio in plasma and CSF, a well‐established biomarker of AD progression [174, 175, 176]. Notably, several APOE receptors, including LDLR, LRP1, VLDLR, and APOER2, also function as receptors for Aβ. This shared receptor system suggests a potential mechanistic link between APOE‐mediated lipid metabolism and Aβ clearance pathways in AD pathogenesis [9].

Neuronal LRP1 clears APOE by endocytosing Aβ/APOE complexes, exhibiting isoform‐dependent efficiency (APOE2 > APOE3 > APOE4) that correlates with AD risk [177]. Aβ can bind to LRP1 either directly or indirectly via physiological ligands, including APOE, α2‐macroglobulin, and lactoferrin. Through this mechanism, Aβ‐containing complexes undergo LRP1‐mediated endocytosis and are cleared from the ISF. LRP1‐mediated transcytosis across the BBB subsequently clears Aβ into the CSF and peripheral circulation [173]. The influence of APOE on AD pathophysiology exhibits strong isoform dependence. Substantial evidence indicates that APOE ε2 and ε3 isoforms demonstrate significantly higher binding affinity for Aβ compared to the ε4 variant, which may contribute to their differential effects on AD risk and progression [15, 178]. The APOE ε4 isoform exhibits reduced binding affinity for Aβ, leading to impaired clearance and subsequent accumulation of Aβ in the brain. Impaired Aβ clearance mediated by the ε4 allele represents a fundamental mechanism contributing to its pathogenicity in AD [33]. Consequently, the APOE ε2 allele is recognized as a protective genetic factor against AD, demonstrating significantly reduced risk compared to both the ε3 and ε4 variants [9]. Consistent with these findings, both animal and human studies demonstrate that APOE ε4 accelerates Aβ pathology onset and impairs synaptic integrity. Specifically, APOE ε4 carriers (both transgenic mice and humans) exhibit earlier Aβ deposition, reduced synaptic spine density, decreased synaptic protein expression, and impaired glutamatergic signaling: a pathway that is crucial for synaptic plasticity [179, 180, 181]. Collectively, these findings establish APOE ε4 as a major pathogenic driver in AD, orchestrating both its initiation and progression through multiple synergistic mechanisms, including accelerated amyloidogenesis, synaptic dysfunction, and impaired neuronal signaling.

## 6 Aβ Interaction With Lipid Rafts

Recent evidence increasingly supports an alternative hypothesis of AD pathogenesis that highlights the central role of lipid rafts [182, 183]. Lipid rafts are dynamic membrane nanodomains defined by their high enrichment of cholesterol, sphingolipids, and scaffolding proteins. They function as signaling platforms that transduce extracellular cues by linking transmembrane proteins to the cytoskeleton and extracellular matrix [184]. Lipid rafts are also present in intracellular membranes, including the TGN, recycling endosomes [185], and mitochondria‐associated endoplasmic reticulum membranes (MAMs) [186]. Lipid rafts contain the key components for Aβ production—APP and secretases. Furthermore, they serve as a platform where Aβ can interact with APOE and Tau, thereby promoting the aggregation of Aβ oligomers and hyperphosphorylated Tau [187]. A central theory of the lipid raft hypothesis of AD is that the APP cleavage product C99—not Aβ—is the primary pathogenic agent, acting through its role in regulating cholesterol metabolism [183]. Research also shows that genetic, dietary, and age‐related mechanisms disrupt cholesterol homeostasis, contributing to the pathogenesis of AD [188, 189]. The accumulation of intracellular cholesterol elevates C99 levels and upregulates MAM function. Consequently, the upstream functional cause of AD is this enhanced MAM activity, which directly drives the formation of hallmark pathological features, including Aβ plaques and NFTs [186].

As a main component of lipid rafts, cholesterol is crucial for incorporating APP into these microdomains, thereby significantly impacting its processing and the production of Aβ [190]. According to research, the alteration in the brain's lipid composition such as cholesterol and sphingolipids impairs not only cellular communication but also increases inflammation and oxidative stress, ultimately promoting the accumulation of Aβ and the development of AD [191]. Accumulating evidence also indicates that the binding of Aβ peptides to gangliosides in lipid rafts—a process facilitated by cholesterol—enables their insertion into the lipid bilayer and the formation of Aβ oligomers. These oligomers form calcium‐permeable pores in the plasma membrane, disrupting intracellular calcium homeostasis and ultimately leading to the memory impairment and neuronal loss characteristic of AD [192]. Taken together, emerging evidence is challenging the traditional paradigm that links AD solely to the accumulation of Aβ and NFTs. Clarifying the pathogenesis of AD therefore requires further investigation into novel areas, such as cholesterol homeostasis and lipid raft composition. A particular priority is to characterize the complex interplay between APP, lipid rafts, AICD‐binding proteins, and Aβ production, including their bidirectional effects on lipid raft structure.

## 7 Interplay Between Aβ and Tau in AD

Tau protein is a member of the microtubule‐associated protein (MAP) family, encoded by the *MAPT* gene on chromosome 17. It is predominantly expressed in neurons [193], with lower levels in astrocytes and oligodendrocytes [4, 17]. Historically, the Aβ and Tau cascade hypotheses have been the central frameworks for understanding AD pathogenesis. However, therapeutic interventions targeting Aβ or Tau individually have yet to yield significant breakthroughs [194]. Aβ and Tau exhibit a substantial reciprocal relationship, with significant crosstalk in their signaling pathways [17, 195, 196]. In transgenic mice harboring APP, Tau, and presenilin1 mutations, Aβ deposition precedes the development of NFT pathology [197]. On the other hand, immunotherapy studies have demonstrated that reducing Aβ levels can prevent both Tau pathology development and memory deficits [198]. These findings suggest that Aβ acts as a trigger for Tau pathology and that targeting its production may mitigate subsequent Tau‐induced disease progression.

**FIGURE 6:**
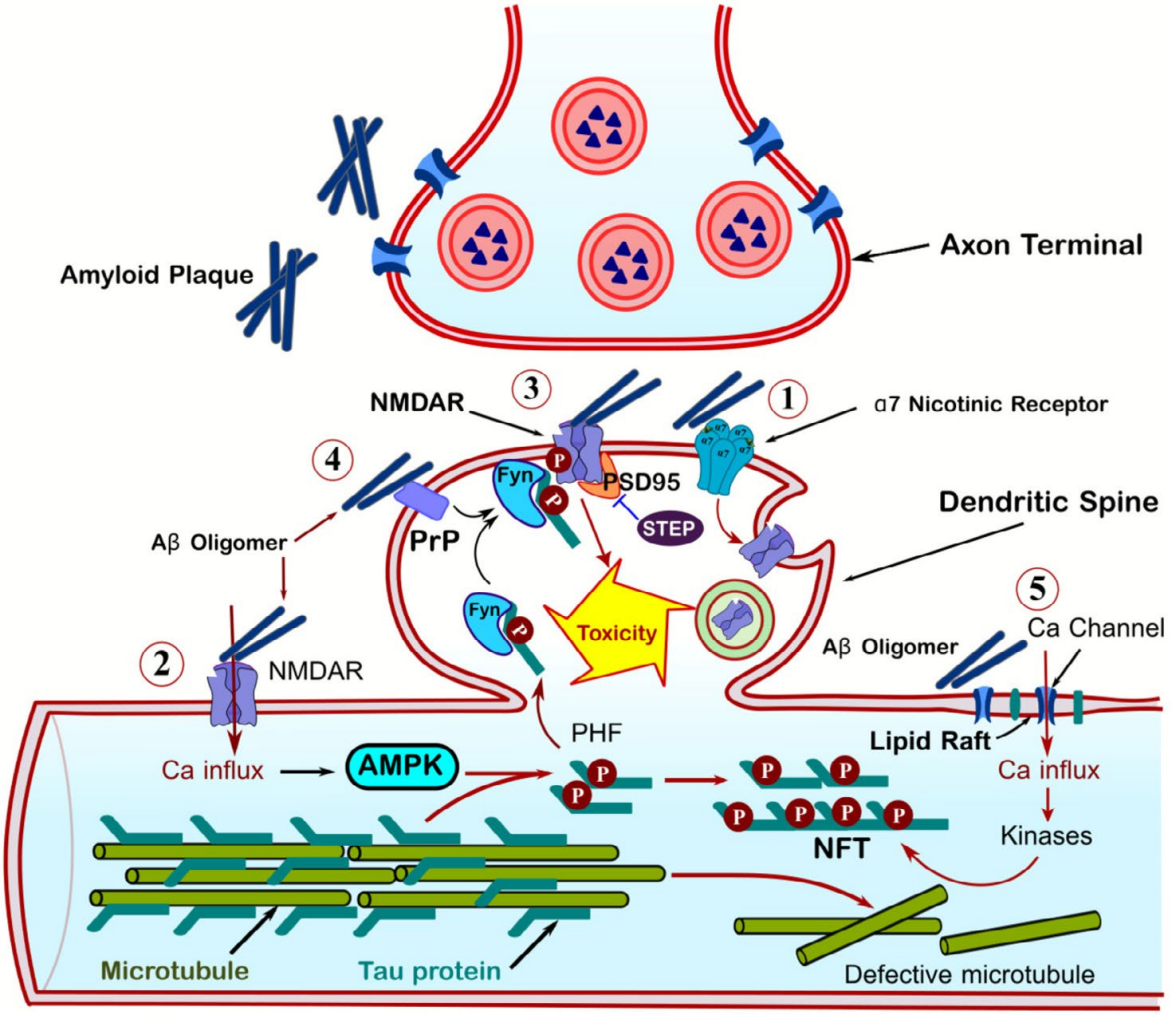
Effects of Aβ oligomers on the structure and function of dendritic spines. (1) Aβ oligomers and protofibrils bind to the α7 nicotinic acetylcholine receptor, triggering NMDA receptor internalization. This disrupts the synaptic–extrasynaptic NMDA receptor balance, shifting activity toward extrasynaptic NMDA receptors. (2) Aβ‐induced extrasynaptic NMDA receptor activation leads to calcium influx, which activates AMP kinase. The kinase then phosphorylates dendritic Tau, detaching it from microtubules and enhancing its binding to Fyn. Consequently, the Tau–Fyn complex migrates to dendritic spines. (3) Within dendritic spines, Fyn phosphorylates the NMDA receptor and facilitates its interaction with PSD95—a key step in Aβ toxicity. Elevated Aβ levels cause excessive NMDA receptor activation, exacerbating downstream toxicity. (4) Aβ also binds directly to prion proteins (PrP), further activating Fyn and promoting NMDA receptor phosphorylation. (5) Additionally, Aβ interacts with the plasma membrane's lipid raft region, inducing calcium influx and activating kinases that drive Tau hyperphosphorylation.

Aβ and Tau mediate synaptic toxicity through multiple mechanisms. For instance, Aβ exerts its effects by preferentially binding to lipid raft microdomains. The binding of Aβ to lipid rafts induces calcium influx and activates downstream kinases, including those implicated in Tau hyperphosphorylation. As Aβ oligomers accumulate, they progressively disrupt lipid raft‐mediated signal transduction through two key mechanisms, including physical obstruction of membrane protein mobility and dysregulation of calcium‐dependent signaling cascades. Additionally, through its interaction with α7 nicotinic acetylcholine receptors (α7nAChRs), Aβ promotes NMDA receptor internalization from dendritic spines. The resulting impairment of calcium signaling initiates a degenerative cascade culminating in spine shrinkage and synaptic dysfunction [114] (Figure 6).

Calcium influx through extrasynaptic NMDARs activates AMPK, which phosphorylates dendritic Tau. Phosphorylated Tau dissociates from microtubules and binds Fyn kinase, forming a complex that translocates to spines. Here, Fyn phosphorylates NMDAR GluN2B subunits, facilitating PSD95 recruitment. This maladaptive signaling complex both mediates Aβ toxicity and promotes excitotoxic synaptic loss through hyperactivated NMDARs [17, 199]. The involvement of Fyn kinase in mediating Aβ oligomer synaptotoxicity was first demonstrated in 1998 [200]. Transgenic AD mouse models demonstrate that Fyn kinase overexpression accelerates both synaptic loss and cognitive decline, highlighting its crucial role in disease progression [201, 202]. Notably, Fyn‐knockout AD transgenic mice (Tg/fyn−/−) show a significant reduction in both Tau accumulation and Tau‐induced neuropathology, further supporting Fyn's critical role in AD pathogenesis [203]. These results establish Fyn kinase as a critical connection between Aβ and Tau pathology, with dual therapeutic implications, including both targeting upstream (Aβ‐Fyn interaction) and downstream (Fyn‐Tau signaling) for both AD and primary tauopathies [203, 204]. Together, the formation of NFTs from hyperphosphorylated Tau underscores the critical role of protein kinases in AD. Consequently, inhibiting these kinases to reduce Tau hyperphosphorylation represents a promising strategy for diagnosing and treating tauopathies [205, 206].

Site‐specific Tau phosphorylation modulates Aβ oligomer toxicity through distinct mechanisms. Notably, p38 MAPK‐mediated phosphorylation at Thr205 inhibits the formation of the Tau‐Fyn‐PSD95‐NMDA receptor complex and subsequent Aβ oligomer‐induced neurotoxicity [207]. Aβ oligomers bind with high affinity to cellular prion protein (PrPc), triggering Fyn kinase activation, pathological NMDA receptor phosphorylation, and downstream synaptic dysfunction [208]. In AD progression, accumulating Aβ activates STEP (STriatal‐Enriched protein tyrosine Phosphatase), which dephosphorylates and inactivates Fyn kinase. This Aβ‐STEP‐Fyn axis drives dendritic spine collapse, synaptic elimination, and cognitive decline [209, 210]. Aβ accumulation starts a pathogenesis cascade that results in NFT formation as well as other pathogenic factors such as apoptotic cascades that may result in wide neuronal damage, cell death, and clinical symptoms associated with AD. Therefore, reducing Tau levels via preventing Tau/Fyn or NMDAR/PSD95 interaction may be a proper strategy to downstream signaling of excitotoxic NMDA receptors (Figure 6). Collectively, these findings support a dual‐hit mechanism of AD pathogenesis, wherein Aβ acts as the trigger that initiates synaptic dysfunction and pathological cascades, while Tau serves as the executor that mediates neurotoxicity and propagates damage [99].

## 8 Aβ as a Biomarker in AD

There is no single, definitive assay for diagnosing AD. Instead, a variety of manual, automated, and semi‐automated techniques have been developed and utilized to measure whole and regional brain volumes, tissue morphology, and rates of atrophy in AD patients, individuals with MCI, and age‐matched healthy controls [211]. Current diagnostic approaches for AD rely on a combination of methods, including the Mini‐Mental State Examination (MMSE), measurements of Aβ and Tau levels in the blood and CSF, structural brain imaging via magnetic resonance imaging (MRI), and positron emission tomography (PET) scanning to assess glucose metabolism in the brain [212, 213]. Recent advances in biofluid phosphorylated Tau (p‐Tau) assays and improvements in p‐Tau tracers have also provided promising insights for earlier disease detection [214]. The combined use of these methods enables more accurate diagnostic assessments. Currently, biomarkers with higher specificity and sensitivity allow for early detection of AD in preclinical stages, facilitating more effective prediction of disease progression.

Biomarkers play a critical role in both monitoring patients with MCI at risk of AD progression and assessing the efficacy of novel therapeutic interventions [215, 216]. Substantial evidence supports that Aβ plaque formation precedes NFT development. Since amyloid deposition progresses over years to decades during the preclinical stage, detecting and measuring Aβ oligomers has proven clinically valuable as an early indicator of AD pathology [217]. In a recent report, researchers used a highly sensitive immunoassay to measure lecanemab‐associated Aβ protofibril levels in CSF, finding a strong correlation with both Aβ plaque burden and neurodegeneration severity [218]. When compared to age‐matched cognitively normal controls, CSF analysis in AD patients exhibits decreased Aβ42/Aβ40 ratio, increased total Tau (t‐Tau) and phosphorylated Tau (p‐Tau) levels, and elevated p‐Tau/Aβ42 ratio (the most accurate CSF diagnostic biomarker combination) [215, 219]. Isoprostane and homocysteine represent two additional biomarkers that have been investigated in CSF. Isoprostanes are commonly used as markers of oxidative stress and lipid peroxidation, processes associated with various diseases including neurodegenerative disorders like AD. Similarly, elevated homocysteine levels are considered a potential risk factor for AD development [217, 220]. Several studies have reported elevated levels of both isoprostanes and homocysteine in individuals with AD, further supporting their potential role as biomarkers in AD pathogenesis [221, 222].

In addition to CSF biomarkers, recent research has identified novel markers associated with axonal damage and synaptic dysfunction. These include granin, SNAP‐25, synaptotagmin, and the calcium‐sensing protein visinin‐like protein 1 (VLP‐1). These biomarkers are particularly promising as they can be detected in the early stages of disease progression [223]. Chitinase‐3‐like protein 1 (CHI3L1/YKL‐40), a glial activation marker expressed in both microglia and astrocytes, along with TREM2, a microglia‐specific biomarker, shows potential for both AD diagnosis and therapeutic monitoring. These neuroinflammatory markers may provide valuable insights into disease progression and treatment response [224]. As previously established, older individuals exhibiting comparable levels of Aβ deposition demonstrate heterogeneous cognitive performance profiles, a variability strongly associated with differences in cognitive reserve capacity [225]. Research indicates that cognitive reserve operates primarily by delaying the clinical manifestation of symptoms rather than attenuating the underlying rate of neurocognitive decline [226]. Consequently, cognitive reserve represents an essential factor in assessing cognitive function during the preclinical stage of AD, independent of neuroimaging findings or CSF biomarker profiles [227].

Furthermore, preclinical research indicates that blood‐based biomarkers (BBMs) are a hot area for early AD detection [228]. Kubota et al. [229] assessed plasma biomarkers including Aβ42/40, phosphorylated Tau (p‐Tau181 and p‐Tau217), glial fibrillary acidic protein (GFAP), and neurofilament light chain (NfL), individually and in combination in a Japanese cohort. They found that plasma biomarkers, Aβ42/40 and p‐Tau217, and particularly their ratio (p‐Tau217/Aβ42), show strong potential as Aβ PET alternatives for AD diagnosis. Grande et al. recently reported only weak associations between blood Aβ42/40 ratio and incident dementia due to AD. Therefore, several attentions are necessary before plasma Aβ can be used to predict AD. First, Aβ concentrations in blood are substantially lower—up to tenfold—than in the CSF. Second, Aβ molecules, particularly Aβ42, are produced in substantial quantities in peripheral organs such as the liver, skeletal muscle, and vascular tissue. Finally, mass spectrometry offers greater accuracy for quantifying blood levels of Aβ, but it is difficult to scale and unaffordable in community settings [230]. Furthermore, non‐coding RNAs are emerging as promising BBMs for the diagnosis and treatment of AD [231, 232]. Together, it seems that using BBMs along with clinical practice will simplify the diagnostic process of AD and simplify timely access to treatments for AD patients at the early phase of the disease [233]. Substantial challenges remain in the practical implementation of BBMs for early detection of AD, necessitating greater efforts to promote their application, establish diagnostic thresholds, and fully elucidate their clinical significance [234, 235].

Recent advances in neuroimaging techniques now enable the quantification of functional connectivity in the aging brain during neural activation using functional MRI (fMRI) [236]. fMRI enables measurement of both task‐related regional brain activity (during motor, sensory, or cognitive tasks) and resting‐state functional connectivity through detection of blood oxygen level‐dependent (BOLD) signals reflecting localized changes in cerebral blood flow and oxygenation [237]. Brain atrophy‐induced changes in cerebral metabolism and hemodynamics may significantly alter BOLD signal characteristics in fMRI studies. Additionally, magnetic resonance spectroscopy (MRS) is a non‐invasive neuroimaging technique that quantifies alterations in key neurochemical metabolites, particularly N‐acetylaspartate (NAA) and myo‐inositol (mI), in AD and MCI patients relative to cognitively normal controls. AD patients demonstrate significantly decreased NAA concentrations and elevated mI levels compared to age‐matched healthy controls, with these metabolic alterations being most pronounced in the temporal lobe and hippocampal regions [238, 239].

Furthermore, PET imaging utilizes specific radiolabeled tracers targeting both Aβ plaques and NFTs in AD. Notable Aβ‐binding radiotracers including Pittsburgh compound B (PiB) and florbetapir (18F) selectively bind to fibrillar and diffuse amyloid plaques, enabling detection of both AD and CAA [240, 241]. Fluorodeoxyglucose (^18^F‐FDG) serves as a widely used radiolabeled tracer for assessing cerebral glucose metabolism in PET imaging. Beyond metabolic measurements, PET neuroimaging enables quantification of neurotransmitter systems, enzyme activity patterns, and neuroinflammatory processes through the administration of specialized receptor‐targeting radioligands [242]. Collectively, the three most reliable neuroimaging biomarkers of AD include: (1) medial temporal lobe atrophy visualized on structural MRI, (2) hypometabolism in the posterior cingulate and temporoparietal cortices detected by ^18^F‐FDG PET, and (3) cortical Aβ deposition identified through amyloid PET imaging [224].

## 9 Therapeutic Strategies for AD Targeting Aβ

Historically, therapeutic approaches for AD have fallen into three main categories: (1) symptomatic treatment targeting general cognitive manifestations, (2) management of neuropsychiatric symptoms, and (3) disease‐modifying therapies. Current pharmacological interventions for symptom management include memantine, an NMDA receptor antagonist, and cholinesterase inhibitors, which are primarily used to mitigate global cognitive symptoms [243]. The second therapeutic category comprises psychotropic medications, including anticonvulsants (e.g., valproate), antidepressants (e.g., selective serotonin reuptake inhibitors or SSRIs), atypical antipsychotics (e.g., risperidone). These agents are primarily employed for managing neuropsychiatric symptoms in AD patients [244]. The third group of medications targets diverse disease‐modifying pathways, including zinc and copper regulators, natural antioxidants such as vitamin E and curcumin, *Ginkgo biloba* extracts, and omega‐3 fatty acids. This category also encompasses β‐secretase and γ‐secretase inhibitors along with α‐secretase activators, agents inhibiting Aβ and Tau aggregation, and compounds blocking Aβ‐activated signaling pathways involving GluR, Fyn, GSK3β, and CDK5 [1, 245]. Additionally, it has been shown that vitamin D regulates inflammatory mediators and antioxidants, resulting in neuroprotective effects, and also impacts neurotransmitter synthesis and brain plasticity [246, 247]. Therefore, elucidating the molecular mechanisms by which vitamin D exerts its protective effects against AD symptoms is a key objective for future research.

The cholinergic deficit in AD was first identified in 1970, when researchers discovered significantly reduced acetylcholine levels in patients' brains. Over the past few decades, therapeutic strategies have predominantly focused on augmenting cholinergic neurotransmission, particularly through acetylcholine enhancement, as a means to improve memory function. The cholinergic hypothesis (one of the fundamental AD hypotheses alongside the Aβ cascade, tauopathy, and neuroinflammation hypotheses) posits that acetylcholine plays an essential role in learning and memory processes [248, 249, 250]. Impaired neurotransmission at cholinergic synapses, along with disrupted neuronal signaling, contributes to the behavioral and cognitive dysfunction observed in AD patients [251]. Experimental studies have shown that intravenous infusion of young blood plasma in AD mouse models rescues learning and memory deficits by functionally reconstituting hippocampal cholinergic networks. This neurorestorative effect appears to be mediated through the reactivation of synaptic plasticity mechanisms in the septo‐hippocampal pathway [252]. Moreover, the novel T‐type calcium channel enhancer SAK3 demonstrates dual therapeutic potential in AD models, showing both cognitive‐enhancing effects and reduction of Aβ accumulation. Its mechanism of action involves enhanced hippocampal acetylcholine release and promotion of neurogenesis, suggesting a multi‐target approach to AD treatment [253]. Consequently, cholinergic signaling remains a critical therapeutic target in AD, forming the basis for currently approved pharmacological treatments.

More advanced approaches in treating AD include gene therapy and immunotherapy strategies [254, 255]. Research indicates that viral delivery of Neurotrophic Factor‐α1/Carboxypeptidase E (NF‐α1/CPE) in the hippocampus prevented the later development of cognitive deficits in mice, supporting the promise of gene therapy for AD [256]. Although animal studies of gene therapy targeting neurotrophins have shown valuable results, still there are no promising outcomes in human trials [257]. In summary, the main challenges in AD gene therapy include better carrier molecules, improved delivery methods, and new therapeutic targets [254]. Researchers believe that vaccines (active immunization) or specific antibodies (passive immunization) that aim to promote Aβ clearance are a promising strategy for treating AD [258]. In active immunotherapy for AD, using vaccines like AN1792, Amilomotide, and UB‐311, the immune system is stimulated by administering Aβ or its fragments, leading to the long‐term production of endogenous antibodies. Although this approach can yield high antibody concentrations with fewer injections and lower medical costs, its associated side effects and adverse immune reactions are difficult to control [255, 258].

The failures of active immunotherapy prompted a shift in research focus toward passive immunotherapy using humanized monoclonal antibodies against Aβ. Several anti‐Aβ monoclonal antibodies have been developed during recent years, most of which are still in their clinical trials. The first FDA‐approved antibody was Aducanumab, a human IgG1 monoclonal antibody, that targets soluble oligomers and insoluble fibrils to prevent the formation of Aβ plaques. Donanemab, Lecanemab, Solanezumab, Gantenerumab, and others are newer, developing anti‐Aβ antibodies, each with a specific affinity for various parts of the Aβ peptide. All of these antibodies are currently in Phase III clinical trials, although the FDA has also granted accelerated approval to donanemab and lecanemab, similar to aducanumab [259, 260]. The specificity and affinity of antibodies for their targets have made them a successful approach in AD treatment. Several mechanisms have been proposed for antibody‐mediated immunotherapy. First, anti‐Aβ antibodies cross the BBB and bind to insoluble Aβ, dissolving Aβ plaques. This may prevent Aβ seeding in the brain. Second, microglia become activated and internalize the anti‐Aβ antibody complex. Third, neurons endocytose the antibody‐target complex, which is then cleared via lysosomal degradation [261]. According to research, the adverse effects of anti‐Aβ antibodies can be managed more easily than those of vaccines due to their targeting of specific protein conformations. However, the need for repeated dosing and higher costs are the main disadvantages of passive immunotherapy. Additionally, active and passive immunotherapy may both result in hyperactivation of the innate and adaptive immune responses, which can consequently induce adverse side effects in the brain [262, 263]. Therefore, future research must aim to enhance the efficacy and minimize the adverse effects of anti‐Aβ antibody immunotherapy for AD.

A major complication for anti‐Aβ antibody immunotherapy is crossing the BBB to enter the brain. Improving drug delivery mechanisms across the BBB is therefore of great importance. Researchers are working on several strategies, including focused ultrasound (FUS) with microbubbles, receptor‐mediated transcytosis (RMT), and nanoparticle carrier systems, to improve the delivery of treatments to the brain [264]. Studies show that anti‐Aβ antibodies engineered with cell‐penetrating single‐chain variable fragments (scFv) can bind the transferrin receptor (TfR) to facilitate transit across the BBB in mice [265]. Pizzo et al. [266] recently developed an antibody transport vehicle that targets the transferrin receptor to deliver Aβ antibodies to the brain. They demonstrated that their method significantly reduced amyloid‐related imaging abnormalities (ARIA) and improved plaque target engagement in a mouse model of amyloid deposition. Researchers also found that a nanomicelle‐mediated delivery system enables anti‐Aβ antibody fragments to cross the BBB, leading to reduced levels of toxic Aβ species and inhibited plaque formation in a mouse model of AD [267]. However, a critical need remains to improve BBB penetration and enhance the efficacy of existing treatments while minimizing their side effects, in parallel with developing novel therapeutic agents.

## 10 Conclusion

AD is a complex condition resulting from a combination of genetic and environmental risk factors. Some of these factors disrupt gene expression, which leads to the production of harmful molecules, triggers inflammation, and initiates other processes that accelerate aging. These processes collectively contribute to the characteristic neuropathology of AD, including the accumulation of Aβ plaques and NFTs. Although Aβ has been central to AD pathogenesis, recent experimental and clinical evidence has challenged this paradigm, and its status as the primary cause of sporadic AD is now debated. Aβ appears to reside within a central signaling pathway that drives neurodegeneration in AD. However, without a clear map of its upstream regulators and downstream effectors, it is impossible to fully unravel the molecular mechanisms that underlie disease pathogenesis and hinder effective diagnosis and treatment. Consequently, the roles of neuroinflammation, mitochondrial dysfunction, oxidative stress, PTMs of Aβ, altered lipid raft composition, and disrupted cholesterol metabolism must now be integrated into a revised model of AD pathogenesis, diagnosis, and treatment.

## Author Contributions

S.A., S.K., and K.A.: Conceptualization and design of the work, writing – original draft preparation, and figures designing. K.H.: Writing – reviewing and editing. All authors approved the final version of the manuscript for publication.

## Funding

The authors received no specific funding for this work.

## Ethics Statement

The authors have nothing to report.

## Conflicts of Interest

The authors declare no conflicts of interest.

## Data Availability

The authors have nothing to report.
